# Essential role of STAT5a in DCIS formation and invasion following estrogen treatment

**DOI:** 10.18632/aging.103586

**Published:** 2020-07-31

**Authors:** Sundee Dees, Laura Pontiggia, Jean-Francois Jasmin, Federica Sotgia, Michael P. Lisanti, Isabelle Mercier

**Affiliations:** 1 Department of Pharmaceutical Sciences, Philadelphia College of Pharmacy, University of the Sciences, Philadelphia, PA, USA; 2 Department of Mathematics, Physics and Statistics, Misher College of Arts and Sciences, University of the Sciences, Philadelphia, PA, USA; 3 Translational Medicine, School of Science, Engineering and Environment (SEE), Biomedical Research Centre (BRC), University of Salford, Greater Manchester, United Kingdom; 4 Program in Personalized Medicine and Targeted Therapeutics, University of the Sciences, Philadelphia, PA, USA

**Keywords:** STAT5a, DCIS, estrogen, invasion, Cav-1

## Abstract

Ductal carcinoma in situ (DCIS) is one of the earliest stages of breast cancer (BCa). The mechanisms by which DCIS lesions progress to an invasive state while others remain indolent are yet to be fully characterized and both diagnosis and treatment of this pre-invasive disease could benefit from better understanding the pathways involved. While a decreased expression of Caveolin-1 (Cav-1) in the tumor microenvironment of patients with DCIS breast cancer was linked to progression to invasive breast cancer (IBC), the downstream effector(s) contributing to this process remain elusive. The current report shows elevated expression of Signal Transducer and Activator of Transcription 5a (STAT5a) within the DCIS-like lesions in Cav-1 KO mice following estrogen treatment and inhibition of STAT5a expression prevented the formation of these mammary lesions. In addition, STAT5a overexpression in a human DCIS cell line (MCF10DCIS.com) promoted their invasion, a process accelerated by estrogen treatment and associated with increased levels of the matrix metalloproteinase-9 (MMP-9) precursor. In sum, our results demonstrate a novel regulatory axis (Cav-1♦STAT5a♦MMP-9) in DCIS that is fully activated by the presence of estrogen. Our sudies suggest to further study phosphorylated STAT5a (Y694) as a potential biomarker to guide and predict outcome of DCIS patient population.

## INTRODUCTION

According to the American Cancer Society, an estimated 250,000 women will be diagnosed with breast cancer (BCa) this year alone and more than 40,000 women will succumb to this devastating disease. Ductal carcinoma in situ (DCIS) is one of the earliest stages of BCa in which cancerous epithelial cells proliferate within the basement membrane of the breast milk duct. Importantly, DCIS lesions are associated with an increased risk of progressing to invasive breast cancer (IBC) over time [1–4]. Strikingly, 50% of DCIS recurrence presents as invasive cancer [5], thus understanding the molecular pathways involved in the development and progression of these pre-invasive lesions is key in bringing BCa to a halt at its early stage [1, 2, 5, 6].

Caveolin-1 (Cav-1) is an important 22-kDa integral membrane protein and structural component of caveolae implicated in the compartmentalization of signaling molecules [7, 8]. Cav-1 has been reported to positively and negatively impact tumor growth through tissue-specific functions [9]. Although oncogenic properties of Cav-1 have been demonstrated in BCa, evidence suggests a tumor suppressive role of Cav-1 in BCa [7, 10]. There is increasing attention to a specific role of Cav-1 in the tumor-associated stroma. A loss of Cav-1 expression in the breast tumor stroma correlated with an increased risk for early recurrence, metastatic progression, and decreased survival in patients [11–13]. A retrospective patient cohort study revealed that nearly 90% of estrogen receptor (ER) positive DCIS patients that had recurred to IBC showed diminished or completely absent Cav-1 expression in their tumor stroma [14]. Additional studies also demonstrated that loss of stromal Cav-1 expression coupled with the gain of stromal monocarboxylate transporter 4 (MCT4) expression was implicated in the transition of DCIS to IDC [15]. However, very little is currently known about the underlying mechanisms associated with DCIS formation and progression in the context of a Cav-1 negative micro-environment.

Signal transducer and activator of transcription 5a (STAT5a) is a transcription factor implicated in cell cycle progression, invasion, and migration in solid cancers [16, 17]. Hemizygous loss of STAT5a in a tumor-prone WAP-Simian Virus 40 T antigen (TAg) transgenic mouse model significantly delayed mammary cancer progression, as evidenced by a reduction in tumor size and number [18]. Conversely, transgenic overexpression of a constitutively activated STAT5a mutant or its upstream JAK2 activator was sufficient to cause tumor formation in the mammary glands of mice [19–21]. Most recently, a role of STAT5a signaling in pre-cancerous breast lesions was also reported, where hyperprolactinemia-inducing antipsychotics were demonstrated to induce activation of STAT5a and suppress apoptosis [22]. In addition, STAT5a-regulated NOX5-L expression increased the invasion and migration of human SK-BR-3 breast adenocarcinoma cells [23] and the inhibition of STAT5a by N-α-acetyltransferase protein (Naa10p) led to the suppression of cell motility and invasion in human MCF-7 and MDA-MB-231 BCa cells [24]. STAT5a was also shown to promote the progression of human atypical ductal hyperplasia (ADH), a precursor of DCIS [25].

Surprisingly, there is very limited data on the contribution of STAT5a in the context of early BCa, especially DCIS formation and progression to invasive disease. However, knowing that several reports link STAT5a expression to tumor progression, and previous studies have demonstrated an inverted relationship of Cav-1 and STAT5a expression both *in vitro* and *in vivo* [26, 27], the current work focuses on the specific role (s) of STAT5a in early estrogen-stimulated BCa formation and progression, using both our established mouse model of estrogen-induced DCIS lesions in Cav-1 KO mice [28] and *in vitro* human DCIS cells with differing STAT5a expression levels.

Herein, our current report establishes that phosphorylated STAT5a (Y694) expression is significantly upregulated in the epithelia of DCIS lesions in Cav-1 KO mice following estrogen treatment, compared to wild-type (WT) mice. Functionaly, STAT5a deletion in Cav-1 KO mice prevented mammary ductal branching and foci (DCIS-like lesion) formation, reduced the accumulation of PCNA positive epithelial cells, and maintained mammary ductal integrity by exhibiting both normal basement membrane and smooth muscle actin (myoepithelial) layer following estrogen treatment, suggesting that STAT5a could also play a role in invasion. Our results also demonstrate a direct contribution of STAT5a on the invasion of a human DCIS cell line (MCF10DCIS.com), an effect amplified by the presence of estrogen and associated with increased expression of a matrix metalloproteinase-9 (MMP-9) precursor protein levels. Collectively, we propose STAT5a as an important player into both DCIS formation and invasion and its phosphorylation on tyrosine 694 (Y694) should be given closer attention as a potential target to prevent DCIS formation and transition to invasive cancer in a subset of high risk patients such as those with a Cav-1 negative cancer stroma. Having a better understanding of STAT5a pathway in pre-invasive breast cancer could lead to the development of personalized therapies for high risk DCIS patients.

## RESULTS

### Cav-1 KO DCIS lesions display increased phosphorylated STAT5a (Y694) levels as a response to 17β-estradiol treatment

**Figure 1:**
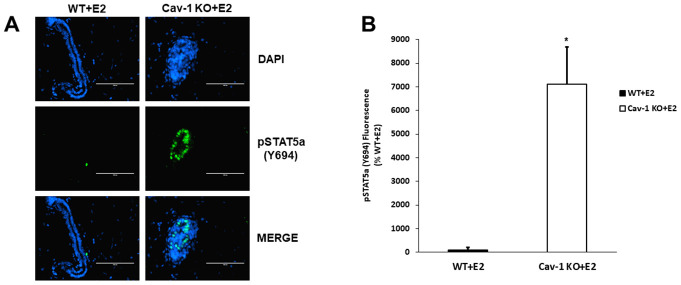
**Cav-1 KO DCIS lesions display increased phosphorylated STAT5a (Y694) levels as a response to 17β-Estradiol treatment.** (**A**) Mammary glands of estrogen-treated WT and Cav-1 KO mice were immunostained with an antibody recognizing phosphorylated STAT5a (Y694). DAPI was used as a nuclear counterstain. The EVOS FL microscope was used to capture images at 40x objective with the DAPI and CY5.5 light cubes (blue: DAPI immunostaining; green: phosphorylated STAT5a (Y694) immunostaining). For each experimental group, immunofluorescence was performed in triplicate on mammary glands derived from 3 independent mice. (**B**) Immunofluorescence staining was quantified using Image J software. Corrected total cell fluorescence (CTCF) was calculated using the following formula: CTCF = Integrated Density – (Area of Selected Region x Mean Fluorescence of Background). Cav-1 KO mammary glands demonstrated a significant increase in phosphorylated STAT5a (Y694) expression compared to estrogen-treated WT counterparts (71.1-fold, p<0.05, n=3). Data are expressed as % WT+E2.

As previously demonstrated, Cav-1 KO mammary glands show hypersensitivity to the proliferative effects of 17β-estradiol (E2) [28]. A similar protocol was utilized to examine if STAT5a was induced during this process and how its deletion could affect DCIS formation in these mice. Immunofluorescence data show that estrogen-treated Cav-1 KO mammary glands exhibit elevated levels of STAT5a (Y694), compared to WT counterparts (Figure 1A) and this increase was quantitatively analyzed as shown in Figure 1B (71.1-fold, p<0.05, n=3 Cav-1 KO vs WT).

### Deletion of STAT5a in Cav-1 KO mice prevents mammary branching and DCIS-Like foci formation

**Figure 2:**
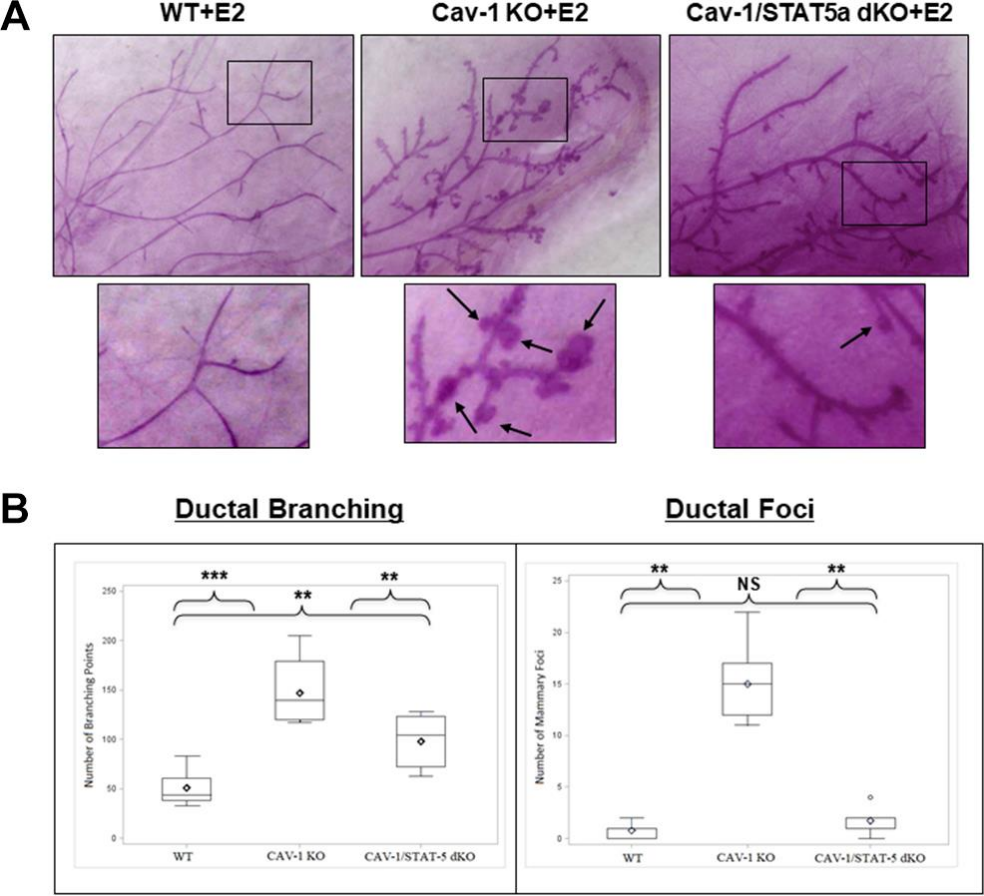
**Deletion of STAT5a in Cav-1 KO mice prevents mammary branching and DCIS-Like Foci formation.** (**A**) Mammary glands of estrogen-treated WT, Cav-1 KO, and Cav-1/STAT5a dKO mice were subjected to whole mount analysis to assess ductal branching and foci formation. Images of the mammary gland whole mounts were captured at 40x objective using an Olympus DP71 camera. Black arrows indicate mammary foci. Original cohort sizes were as follows: WT+E2 (9 mice); Cav-1 KO+E2 (7 mice); Cav-1/STAT5a dKO+E2 (8 mice). (**B**) SAS programming software (version 9.4) was used to generate box plots displaying the number of ductal branching points (left panel) and the number of ductal foci (right panel) for each experimental group. Ductal branching was calculated as a summation of primary, secondary, and tertiary branch points. The absence of STAT5a in the Cav-1 KO mammary gland led to a decrease in both ductal branching and foci formation. Quantitatively, changes in ductal branching were as follows (left panel): WT vs. Cav-1 KO (2.9-fold, p<0.001), Cav-1 KO vs. Cav-1/STAT5a dKO (1.5-fold, p<0.01), WT vs. Cav-1/STAT5a dKO (1.9-fold, p<0.01). Quantitatively, changes in ductal foci were as follows (right panel): WT vs. Cav-1 KO (19.3-fold, p<0.01), Cav-1 KO vs. Cav-1/STAT5a dKO (8.6-fold, p<0.01), WT vs. Cav-1/STAT5a dKO (NS, p=0.164).

Since DCIS-like lesions secondary to estrogen treatment in Cav-1 KO mice showed increased levels of phosphorylated STAT5a (Y694) as shown in Figure 1, we questioned whether this was a bystander or functional effect of this transcription factor. To study this hypothesis, we compared branching and lesion formation in ovariectomized female WT, Cav-1 KO, and Cav-1/STAT5a double knockout (dKO) mice treated with an estrogen regimen for 60 days by performing whole mounts analysis. As shown in Figure 2A and 2B, estrogen-treated Cav-1 KO mice showed a significant increase in ductal branching (summation of primary, secondary, and tertiary branch points) compared to WT counterparts (2.9-fold, p<0.001, n=7-9). A homozygous deletion of STAT5a in Cav-1 KO mice was sufficient to significantly reduce the development of mammary branching compared to Cav-1 KO mice (1.5-fold, p<0.01, n=7-8), but still significantly higher than the WT group (1.9-fold, p<0.01, n=8-9) (Figure 2B: left panel). While mice lacking Cav-1 expression exhibited a significant increase in mammary foci formation following estrogen stimulation compared to WT mice (19.3-fold, p<0.01, n=7-9) (Figure 2B: right panel), this increase was significantly reduced in Cav-1/STAT5a dKO mice (8.6-fold, p<0.01, n=7-8, compared to Cav-1 KO mice) (Figure 2B: right panel) which reached the levels observed in WT mice (NS, p=0.164, n=8-9, WT vs Cav-1/STAT5a dKO).

### Proliferating cell nuclear antigen (PCNA) increase in Cav-1 KO DCIS-Like lesions secondary to estrogen treatment is inhibited by a homozygous STAT5a deletion

**Figure 3:**
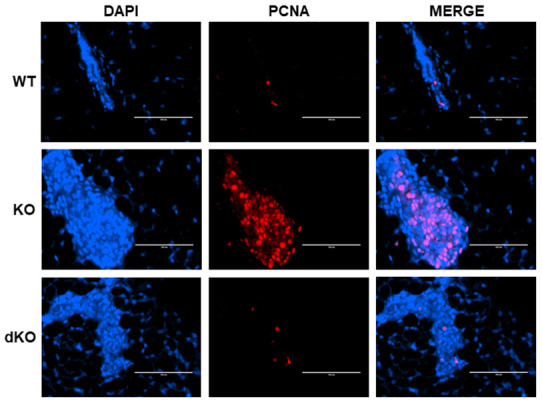
**Proliferating Cell Nuclear Antigen (PCNA) increase in Cav-1 KO DCIS-like lesions secondary to estrogen treatment is inhibited by a homozygous STAT5a deletion.** Mammary glands of estrogen-treated WT, Cav-1 KO, and Cav-1/STAT5a dKO mice were immunostained with an antibody recognizing proliferating cell nuclear antigen (PCNA). DAPI was used as a nuclear counterstain. The EVOS FL microscope was used to capture images at 40x objective with the DAPI and Texas Red light cubes (blue: DAPI immunostaining; red: PCNA immunostaining). Qualitatively, mammary glands lacking Cav-1 expression showed elevated PCNA expression upon stimulation with estrogen compared to WT counterparts. A STAT5a deletion in the Cav-1 KO mammary gland diminished PCNA expression to WT levels. For each experimental group, immunofluorescence was performed in triplicate on mammary glands derived from 3 independent mice.

The data above warranted further exploration into the role of STAT5a in estrogen-induced DCIS lesion formation grown within a Cav-1 negative mammary gland. PCNA, a cofactor of DNA polymerase that functions in the G1 and S phases of the cell cycle [29], was investigated through immunofluorescence in estrogen-stimulated WT, Cav-1 KO, and Cav-1/STAT5a dKO mammary glands (Figure 3). The increased level of PCNA nuclear expression observed in Cav-1 KO compared to WT mammary glands (middle panel) was reduced by a homozygous deletion of STAT5a in Cav-1 KO mice. These observations suggest an implication of STAT5a in the cell cycle progression of Cav-1 KO mammary lesions following estrogen treatment.

### Collagen and smooth muscle actin layer remain uninterrupted in Cav-1/STAT5a dKO mice mammary ducts following estrogen treatment

**Figure 4:**
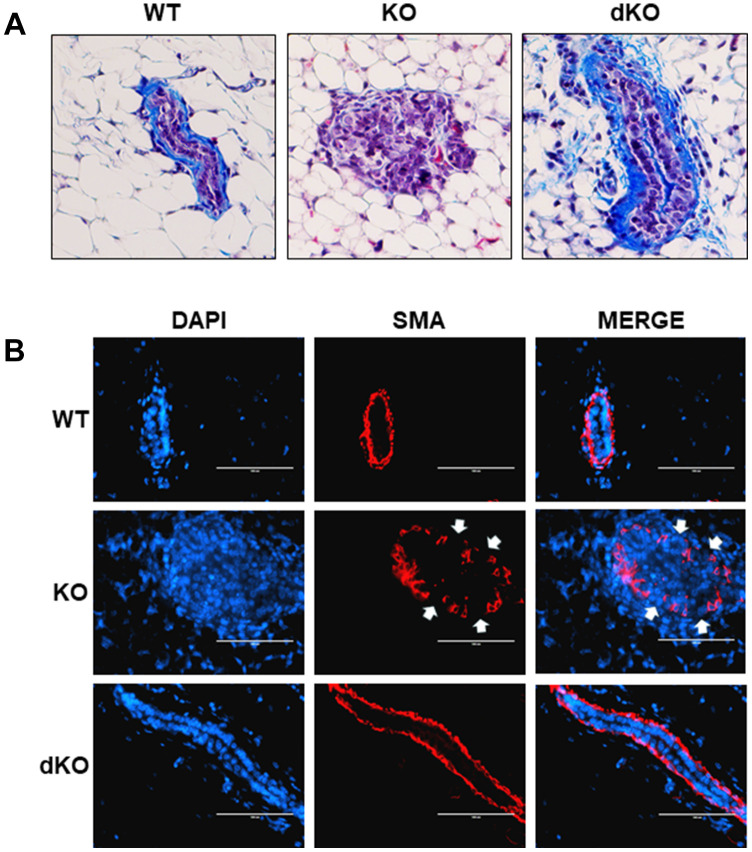
**Collagen and smooth muscle actin layer remain uninterrupted in Cav-1/STAT5a dKO mice mammary ducts following estrogen treatment.** (**A**) Mammary glands of estrogen-treated WT, Cav-1 KO, and Cav-1/STAT5a dKO mice were stained with Masson’s trichrome to highlight the collagen (blue staining) lining the outside of the basement membrane of the ducts. Qualitatively, Cav-1 KO mammary glands stimulated with estrogen demonstrated a complete degradation of collagen surrounding the basement membrane, whereas WT ducts showed intact collagen deposition. Deletion of STAT5a from estrogen-treated Cav-1 KO mice restored collagen deposition to WT levels. For each experimental group, trichrome staining was performed in triplicate on mammary glands derived from 3 independent mice. (**B**) Mammary glands of estrogen-treated WT, Cav-1 KO, and Cav-1/STAT5a dKO mice were immunostained with an antibody recognizing alpha smooth muscle actin (SMA) to highlight the myoepithelial cells lining the inside of the basement membrane of the ducts. DAPI was used as a nuclear counterstain. The EVOS FL microscope was used to capture images at 40x objective with the DAPI and Texas Red light cubes (blue: DAPI immunostaining; red: SMA immunostaining). Qualitatively, the SMA layer surrounding estrogen-treated Cav-1 KO mammary ducts was disrupted (white arrows indicate breaks in the myoepithelial cells), but completely intact around WT ducts. Cav-1 KO mice lacking STAT5a expression maintained an intact SMA layer similar to WT mice. For each experimental group, immunofluorescence was performed in triplicate on mammary glands derived from 3 independent mice.

Given the reported involvement of STAT5a in driving the progression of solid tumors [16–18, 23, 24], we sought to examine closer whether a STAT5a deletion could restore mammary duct structural integrity in Cav-1 KO mice treated with estrogen. While early DCIS lesions are well-contained within the basement membrane of the mammary ducts, later stages of DCIS are characterized by a dismantled basement membrane and the presence of micro-invasion, allowing cancer cells to invade outside into the stroma, a characteristic seen in Cav-1 KO mice treated with estrogen [1, 5, 28]. To assess how STAT5a deletion affected the histological morphology of the mammary ducts in Cav-1 KO mice, we first stained WT, Cav-1 KO, and Cav-1/STAT5a dKO mammary glands with Masson's Trichrome histochemical stain, which highlights collagen layers surrounding the outside of the mammary ducts (Figure 4A). Most intriguingly, collagen deposition (integrity of the basement membrane) was maintained in Cav-1 KO mammary glands lacking STAT5a expression (Figure 4A: right panel), similar to that observed in WT mice following estrogen treatment (Figure 4A: left panel). This is a significant change compared to the degradation of collagen seen around the DCIS lesions of estrogen-treated Cav-1 KO mammary glands (Figure 4A: middle panel). Closely associated with the inner side of the basement membrane is the smooth muscle layer that allows mammary ducts to contract during lactation (myoepithelial layer). This extra layer serves as a barrier that encloses the epithelial cells, preventing their invasion into the stroma [30]. To observe the integrity of the smooth muscle layer, we immunostained WT, Cav-1 KO, and Cav-1/STAT5a dKO mammary glands with an anti-alpha smooth muscle actin (SMA) antibody to visualize the distribution of myoepithelial cells lining the inside of the basement membrane of the mammary ducts (Figure 4B). Consistent with trichrome staining, Cav-1 KO mammary glands lacking STAT5a expression maintained a continuous smooth muscle layer (Figure 4B: bottom panels) similar to WT mammary ducts (Figure 4B: upper panels), compared to Cav-1 KO proliferative lesions which exhibited apparent breaks in the myoepithelial layers (Figure 4B: middle panels). These data suggest a possible role of STAT5a in promoting invasion of Cav-1 KO DCIS lesions.

### Western blot analysis following lentiviral-mediated overexpression of STAT5a in human MCF10DCIS.com

**Figure 5:**
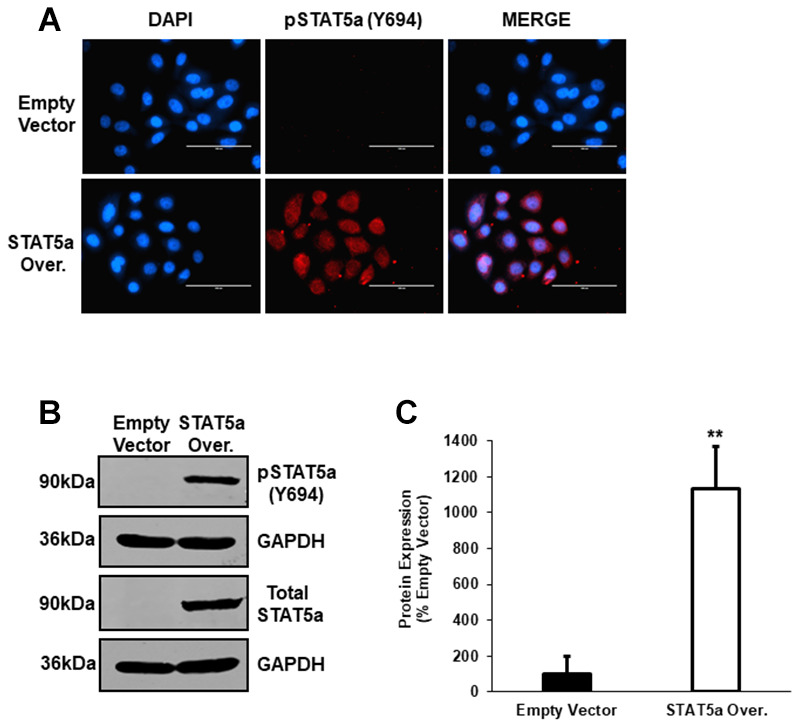
**Western blot analysis following lentiviral-mediated overexpression of STAT5a in human MCF10DCIS.com.** (**A**) Immunofluorescence staining was used to confirm overexpression of phosphorylated STAT5a (Y694) in MCF10DCIS.com cells. Empty vector and STAT5a overexpressor cells were immunostained with an antibody recognizing phosphorylated STAT5a (Y694). DAPI was used as a nuclear counterstain. The EVOS FL microscope was used to capture images at 40x objective with the DAPI and Texas Red light cubes (blue: DAPI immunostaining; red: phosphorylated STAT5a (Y694) immunostaining). Qualitatively, phosphorylated STAT5a (Y694) expression was upregulated in STAT5a overexpressor cells compared to empty vector control cells. Immunofluorescence was performed in triplicate on cells derived from 3 independent passages. (**B**) Western blotting was used to confirm overexpression of phosphorylated STAT5a (Y694) in MCF10DCIS.com cells. Whole cell lysates (100μg) of empty vector and STAT5a overexpressor cells were used to assess the protein expression of phosphorylated STAT5a (Y694) and total STAT5a. GAPDH was used as a control for equal loading. Western blotting was performed in triplicate on cells derived from 3 independent passages. (**C**) Densitometry analysis was performed using the LI-COR imager. A ratio of phosphorylated STAT5a (Y694) to total STAT5a was calculated upon normalizing to respective loading controls. Data are reported as % empty vector. Quantitatively, phosphorylated STAT5a (Y694) expression was upregulated in MCF10DCIS.com STAT5a overexpressor cells compared to empty vector control cells (11.3-fold, p<0.01, n=3).

To further explore the functional role of STAT5a in the progression of 17-β-estradiol-induced DCIS lesions seen in Cav-1 KO mice, we utilized a lentiviral transduction approach to overexpress STAT5a in human MCF10DCIS.com cells. The MCF10DCIS.com cell line is characterized by the presence of high grade, comedo DCIS-like structures and is an ideal model system for studying mechanisms involved in cancer progression [31]. Immunofluorescence staining showed successful nuclear overexpression of phosphorylated STAT5a (Y694) following lentiviral-mediated overexpression compared to empty control vector cells (Figure 5A). This was further confirmed by western blotting (Figure 5B), where densitometry analysis demonstrated a significant increase in phosphorylated STAT5a (Y694) expression in STAT5a overexpressor cells in comparison to empty vector control cells (11.3-fold, p<0.01, n=3) (Figure 5C). Collectively, these data verify the successful overexpression of STAT5a in human MCF10DCIS.com cells.

### STAT5a overexpression in human DCIS cells drives invasion, a phenomenon enhanced by 17-β-estradiol treatment

**Figure 6:**
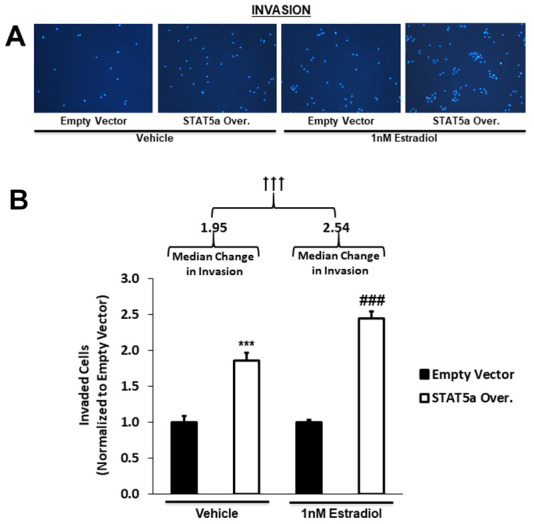
**STAT5a overexpression in human DCIS cells drives invasion, a phenomenon enhanced by 17-β-Estradiol treatment.** (**A**) Qualitatively, transwell invasion assay results depict DAPI-stained invaded cells after 18- hour treatment of MCF10DCIS.com empty vector or STAT5a overexpressor cells with vehicle (DMSO) or 1nM estradiol. Images of the invaded cells were captured using the DAPI channel on the EVOS FL microscope at 10x objective. (**B**) Quantitatively, the number of invaded cells in five representative fields of view were averaged for each membrane. Data were normalized to empty vector. Treatment with vehicle or 1nM estradiol led to a significant increase in invasion of STAT5a overexpressor cells compared to empty vector cells (vehicle-treated empty vector vs. STAT5a overexpressor cells: 1.9-fold, p<0.001, n=3; estrogen-treated empty vector vs. STAT5a overexpressor cells: 2.4-fold, p<0.001, n=3). Moreover, the invasion of STAT5a overexpressor cells relative to empty vector cells was calculated independently for each treatment group and reported as a median change in invasion with an associated interquartile range (IQR) (vehicle [STAT5a overexpressor/empty vector]: 1.95 median, 0.49 IQR, p<0.001, n=3; estrogen [STAT5a overexpressor/empty vector]: 2.54 median, 0.39 IQR, p<0.001, n=3). The median change in invasion of estrogen-treated STAT5a overexpressor cells relative to empty vector cells was significantly higher compared to the median change in invasion of vehicle-treated STAT5a overexpressor cells relative to empty vector cells (p<0.001, n=3).

Qualitative images of invaded cells upon vehicle or estradiol treatment of empty vector vs. STAT5a overexpressor MCF10DCIS.com cells are depicted in Figure 6A. While both vehicle and estrogen-stimulated STAT5a overexpressor cells showed a significant increase in invasion compared to empty vector counterparts (1.9-fold, p<0.001. n=3, vehicle STAT5a overexpressor vs. empty vector) and (2.4-fold, p<0.001, n=3, estrogen STAT5a overexpressor vs. empty vector) (Figure 6B), invasion was significantly higher in the latter (estrogen treated group) (Figure 6B). In fact, the median change in invasion of estrogen-treated STAT5a overexpressor cells relative to estrogen-treated empty vector cells (median of 2.54, IQR of 0.39, n=3) was significantly greater compared to the median change in invasion of vehicle-treated STAT5a overexpressor cells relative to vehicle-treated empty vector cells (median of 1.95, IQR of 0.49, n=3) (p<0.001, n=3) (Figure 6B). These data demonstrate that STAT5a overexpression accelerated the invasion of MCF10DCIS.com cells in an estrogen dependent manner.

### Increased Pro-MMP-9 protein expression secondary to STAT5a overexpression and 17-β-Estradiol treatment suggest a hormonal regulation of DCIS progression with this transcription factor

**Figure 7:**
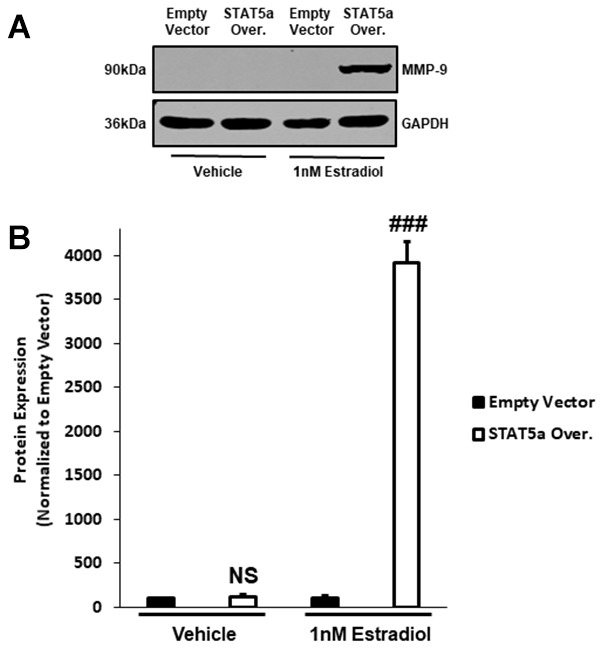
**Pro-MMP-9 protein expression secondary to STAT5a overexpression and 17-β-Estradiol treatment suggest a hormonal regulation of DCIS progression with this transcription factor.** (**A**) MCF10DCIS.com empty vector and STAT5a overexpressor cells were treated with vehicle (DMSO) or 1nM estradiol for 24 hours. Whole cell lysates (75μg) were used to assess the protein expression of MMP-9. GAPDH was used as a loading control. Western blotting was performed in triplicate on cells derived from 3 independent passages. (**B**) Using the LI-COR imager, densitometry was used to determine the protein expression of MMP-9 upon normalizing to the loading control. Data are reported as % empty vector. Quantitatively, no significant difference in MMP-9 expression was observed between vehicle-treated empty vector and STAT5a overexpressor cells (NS, p=0.382, n=3). Upon treatment with estrogen, a significant increase in MMP-9 protein expression was observed in STAT5a overexpressor cells compared to empty vector cells (39.1-fold, p<0.001, n=3).

To further investigate the mechanisms associated with invasiveness of MCF10DCIS.com STAT5a overexpressor cells in response to estrogen, we sought to examine the protein expression of downstream pro-matrix metalloproteinase-9 (MMP-9). Pro-MMP-9 is a precursor that leads to the production of a basement membrane and extracellular matrix endopeptidase degrading enzyme to promote the invasion of cancer cells into the surrounding stroma, and is often used as an index of transcriptional regulation of this gene [32, 33]. Upon treating MCF10DCIS.com empty vector and STAT5a overexpressor cells with vehicle or estrogen, protein expression of pro-MMP-9 was determined by western blot (Figure 7A). Quantitatively, densitometry revealed that while pro-MMP-9 expression did not differ between vehicle-treated empty vector and vehicle-treated STAT5a overexpressor cells (NS, p=0.382, n=3) (Figure 7B), STAT5a overexpressor cells demonstrated a significant increase in protein expression of pro-MMP-9 compared to empty vector counterparts upon estrogen treatment (39.1-fold, p<0.001, n=3) (Figure 7B).

## DISCUSSION

Currently, the molecular mechanisms that govern the transformation of non-invasive DCIS lesions to invasive carcinomas are not well understood [5]. Surgical excision, radiation, and endocrine therapy have become the standard treatment options to reduce the risk of disease progression, even for patients that would otherwise never evolve to IBC [34]. Traditionally, histological grade and hormone receptor status have been used to stratify DCIS patients according to their risk of recurrence and/or progression to IBC [35]. However, these features alone cannot reliably predict which DCIS lesions will progress to an invasive phenotype [1, 35]. As such, there is an unmet need to further understand the molecular pathways involved in DCIS progression.

Studies have reported that an elevated expression of Cav-1 in the stroma of breast cancer patients confers significant protection against progression to metastases and associates with increased chances of survival [11] [36]. In contrast, Hart et al. have attributed a predictive value of Cav-1 expression in the epithelial compartment of invasive breast cancer [37]. Despite Cav-1 having a consistent prognostic value in breast cancer patients, the subtissular compartments associated with these effects seem to differ in the literature. Interestingly, studies focusing on protective effects of Cav-1 expression in the stroma reported its protein levels through stains with a Cav-1 antibody, while the Hart et al. study showing an epithelial protection of Cav-1 expression focused on mRNA levels, which might explain these contrasting observations. Our Cav-1 KO mouse model that develop accelerated DCIS-like lesion formation and invasion secondary to estrogen treatment as previously published, seems to be an appropriate model [28] to uncover the potential mechanisms involved in accelerated DCIS invasion in patients with a lack of stromal Cav-1.

Our current data implicates a functional role of STAT5a in the epithelial compartment of DCIS. Lowering STAT5a levels in estrogen-induced Cav-1 kockout DCIS model in mice prevented lesions formation, restored collagen deposition, maintained myoepithelial cell layer, and decreased PCNA-positive epithelial cells. Although these results are very exciting and might highlight a previously unidentified role of STAT5a in estrogen-induced DCIS formation, our *in vivo* model has certain limitations that should be noted. Firstly, all compartments of the mammary gland of the Cav-1 knockout mice growing these DCIS lesions lack Cav-1 expression (including both stromal and epithelial where the DCIS arise). Future studies will be developed to focus more specifically on the role of STAT5a as it relates to a lack of Cav-1 in the stroma, to closely reproduce the studies in patients [14, 15]. This might be performed by using human CAFs with and without Cav-1 expression that will be co-injected with human DCIS cells to look at STA5a signaling. Another limitation of our current *in vivo* model is that STAT5a was deleted in the mammary glands of Cav-1 KO mice from birth which might also modify how this hormonally regulated tissue might develop and further respond to estrogen to form DCIS lesions. For this specific reason, we set out to examine the role of STAT5a in an *in vitro* model system of already established DCIS using the human MCF10DCIS.com cell line, to better understand the direct contribution of this transcription factor in DCIS progression. MCF10DCIS.com cell line originates from DCIS lesions with comedo growth patterns created by the injection of T24 c-Ha-ras oncogene-transformed MCF10A (MCF10AT) cells into severe combined immune-deficient (SCID) mice. This cell line is an ideal *in vitro* model for studying human DCIS progression [38]. Our current report also demonstrates that STAT5a overexpression in a human DCIS cell causes increased invasion when treated with estradiol. Together our results indicate a possible implication of STAT5a in estrogen-stimulated DCIS progression, a phenomenon that could be initiated by a decrease in stromal Cav-1 *in vivo*, and this possibility will also be tested in future studies with co-cultures with cancer fibroblasts.

To our knowledge, no previous studies have yet implicated STAT5a as an important transcription factor actively involved in DCIS formation and invasion. While several studies ascribe a pro-tumorigenic function of STAT5a in many cancers as described above [16–21], its role in human BCa remains somewhat mixed and controversial. For example, STAT5a expression was predictive of increased overall survival and response to endocrine therapy in ER-positive human invasive breast cancer [39]. In addition, *in vitro* experiments demonstrated that STAT5a inhibited the invasion of well-differentiated ER positive human T-47D cells and poorly differentiated ER negative human BT-20 cells [40]. Taken together, these published reports indicate so far, a dual functionality of STAT5a in human BCa, which seems dependent upon cellular context and the stage of BCa, only to name a few. Although our study demonstrates STAT5a as a key factor during DCIS formation *in vivo* and which also stimulates pro-invasive properties in a DCIS model *in vitro*, more studies will be warented to fully undertand its biological role in early cancer progression. An important result shown in our current report here suggests that estrogen treatment enhances the pro-tumorigenic properties of STAT5a in our DCIS models. So far, very little attention has been given to a potential synergism between STAT5a and estrogen signaling, especially in the context of DCIS formation and progression, and which might also explain differing roles of STAT5a in breast cancer. Furthermore, it appears that paying closer attention to the subtype of estrogen receptor(s) expressed by the cells when STAT5a is present might also be of importance in fully predicting breast cancer cell behaviors. Despite lacking the expression of a traditional nuclear estrogen receptor alpha (ER-α) [41], MCF10DCIS.com invaded significantly more following estrogen treatment when STAT5a was overexpressed. This could be explained by an alternatively spliced variant of full-length ER-α, a shorter ER isoform (ER-α36) and a key mediator of non-genomic estrogen signaling pathways [42, 43] also shown to mediate Scr/EGFR/STAT5 pathway activation and mitogenic effects in ER-negative TNBC cell lines [44, 45]. It could be speculated that the contribution of STAT5a on invasion following estrogen treatment could result from a crosstalk between membrane localized ER-α36 and neighboring growth factor receptors. Important future experiments will focus on overexpressing a traditional nuclear ER-alpha (66KDa) in MCF10DCIS.com and assess whether STAT5a will maintain its pro-invasive properties and/or knocking down ER-alpha 36. Interestingly, a previous study showed that in a model of traditional nuclear ER-alpha-66 overexpression, a deletion of STAT5a did not prevent chemically-induced tumors in the presence of estrogen, which could also hint at the importance of the ER subtypes to fully understand the role of this transcription factor [46]. Also important to mention, is that our current models did not focus on any potential roles or contributions of STAT3, a different related isoform. STAT3 was discovered to have non redundant and sometimes even opposing effects as STAT5a. For example, in T-47D and SK-BR-3 breast cancer cell lines, STAT5a and STAT3 differentially regulated BCL-6 expression, a protein involved in apoptosis [47]. In addition, STAT5a seems to have a dominant effect over STAT3 when co-expressed in the same cell, also a very interesting observation [47]. Although our models herein did not co-express STAT3 and STAT5a or specifically looked at the functional role of STAT3, our future studies will focus on the effects of these 2 transcription factors alone and together in DCIS cell model systems. In sum, clarification of these potential mechanisms of action will be warranted in future pathway exploration studies in human DCIS models.

To further our mechanistic knowledge of the pro-invasive role of STAT5a, we assessed the levels of pro-MMP-9, a pro-enzyme that facilitates degradation and proteolysis of the extracellular matrix to promote cancer cell dissemination and metastasis [32]. Intriguingly, we observed a dramatic upregulation of pro-MMP-9 protein expression in MCF10DCIS.com cells overexpressing STAT5a stimulated with estrogen treatment. The recruitment of this enzyme by STAT5a in DCIS would be consistent with previous studies demonstrating that siRNA and overexpression of MMP-9 modulate invasion, motility and alterations of cytoskeletal components [48]. Our results are also consistent with previous reports confirming a transcriptional regulation of MMP-9 by STAT5a [49]. STAT5a was also previously shown to cause changes associated with epithelial to mesenchymal transition, also consistent with our observation of increased invasion in DCIS cells by this transcription factor [50]. A possible connection between a lack of stromal Cav-1 and an increase in STAT5a in the epithelial DCIS lesion could be through cytokine secretion, through interleukin-6 (IL-6). Interestingly, Cav-1 null mouse fibroblasts have been previously demonstrated to secrete significantly more IL-6 when compared to their wild-type counterparts [51]. Interleukin-6 is a very important cancer cytokine and it is known to be capable of activating the STAT pathway [52]. Whether preventing upstream interleukin-6 secretion by cancer-associated fibroblasts (CAFs) could halt the progression of DCIS lesions to invasive cancer in high-risk DCIS breast cancer patients, such as those with low stromal Cav-1 expression, remains to be elucidated and could have significant clinical impact.

To our knowledge, no studies have yet correlated the levels of STAT5a, especially the Y694 phosphorylated form, to histological grades of DCIS and invasive potential *in vivo* or in patients. Although Shan et al. have previously detected STAT5a in DCIS lesions of chemically-induced rat lesions and human samples, they have not correlated its expression or phosphorylation with histological grade of DCIS [53], therefore this would be a clinically important study to perform and follow up on in a near future. Our future experiments also plan to further our knowledge on the paracrine influence of the stromal cancer compartment on epithelial STAT5a signaling by performing co-culture experiments of CAFs both in *vitro* and in *vivo* also as mentioned above. Our results here thus suggest that there is a potential complex signaling event occurring that could begin in the microenvironment of pre-cancerous patients that could signal and push DCIS lesions to begin invading in some patients and our results point towards a novel regulatory axis (Cav-1♦STAT5a♦MMP-9) that might enable a better understanding of the early events involved in the progression of DCIS lesions to invasive cancers.

In summary, whether phosphorylated STAT5a (Y694) could serve as a clinical biomarker and potential target to help treat Cav-1-depleted DCIS population at greater risk of progressing to IBC remains a future avenue for further exploration. While the presented study is preliminary in nature, we are hopeful that current results will contribute to a better understanding of DCIS progression and potential new avenue of treatment for high risk DCIS patient populations and open up many future research endevours that will focus on this pathway, especially in the context of estrogen stimulation.

## MATERIALS AND METHODS

### Antibodies

An anti-rabbit monoclonal antibody (mAb) against phosphorylated STAT5a (Y694) (Cat#9314S) was purchased from Cell Signaling Technology (Danvers, MA). An anti-mouse mAb against alpha smooth muscle actin (Cat#ab7817) were purchased from Abcam (Cambridge, MA). Anti-mouse mAbs against PCNA (Cat#sc-56) and MMP-9 (Cat#sc-393859) were purchased from Santa Cruz Biotechnology (Santa Cruz, CA). An anti-mouse mAb against glyceradehyde-3-phosphate dehydrogenase (GAPDH) (Cat#10R-2932) was purchased from Fitzgerald Industries International (Acton, MA).

### Mouse model

This study was conducted according to the guidelines of the National Institutes of Health (NIH) and the Thomas Jefferson University Institute for Animal Studies. Approval was granted by the Institutional Animal Care and Use Committee (IACUC) at Thomas Jefferson University. All mice used in this study were in the FVB/N genetic background. Experimental groups included wild type (WT), Cav-1 knockout (KO), and Cav-1/STAT5a double knockout (dKO) mice. WT mice and STAT5a mice were purchased from Jackson Laboratories (Bar Harbor, Maine). Cav-1 KO mice were generated as previously described [54]. To generate the Cav-1/STAT5a dKO mice, Cav-1 KO female mice were bred with STAT5a male mice to generate Cav-1/STAT5a heterozygotes. Then, female and male heterozygotes (Cav-1/STAT5a) were bred to generate Cav-1/STAT5a dKO mice. To maintain the colony, male Cav-1/STAT5a dKO mice were bred with female Cav-1 KO/STAT5a heterozygotes since the complete lack of STAT5a interferes with proper lactation [55]. Genotypes were confirmed by polymerase chain reaction (PCR) as followed by Jackson Laboratories protocol (Bar Harbor, Maine).

### Bilateral ovariectomy procedure

Mice were ovariectomized as previously described [28]. Briefly, 5-week old female WT, Cav-1 KO, and Cav-1/STAT5a dKO mice were anesthetized using 5mg/kg xylazine and 50mg/kg ketamine. A single dorsal incision followed by ligation of the ovarian arteries and veins with a 4-0 silk suture was performed, followed by the excision of both ovaries. The incision site was subsequently closed with a 5-0 silk suture and the mice were administered a subcutaneous injection of analgesic (0.1mg/kg buprenorphine). Mice were allowed to recuperate for 2 weeks before being randomly assigned to 2 replacement pellets containing 17-β-estradiol (7.5 mg/pellet; 60-day slow release; 125 μg/day) or placebo pellets (Innovative Research of America, Sarasota, FL). Implantation of slow-release pellets was performed under anesthesia by lifting the skin on the lateral side of the neck of the mice and by making an incision equal in diameter to that of the pellet. Then, with a pair of forceps, a horizontal pocket of about 2 cm beyond the incision site was created, and the pellet was introduced. The incision site was closed with a 5-0 silk suture.

### Preparation of tissues

Mice were euthanized by inhalation of CO_2_ (compressed CO_2_ gas cylinder) at 60 days following pellet implantation. After the mice were euthanized, the inguinal mammary gland #4 was excised and fixed in formalin for 24 hours, paraffin-embedded, and cut into 5 μm sections for histological analyses.

### Immunofluorescence on tissues

Paraffin-embedded sections of mammary glands were dehydrated in xylene for 20 minutes and rehydrated in a series of graded ethanol solutions and distilled water for 5 minutes each. Tissues were then incubated in a citric acid-based antigen unmasking solution with an acidic pH (Cat#H-3300, Vector Laboratories, Burlingame, CA) using an electric pressure cooker on high pressure for 5 minutes. Tissues were washed three times with Dulbecco’s phosphate buffered saline (DPBS) (1x) for 5 minutes each wash. Then, tissues were blocked with 10% goat normal serum (Cat#S-1000, Vector Laboratories, Burlingame, CA) for 1 hour at room temperature and incubated with a given primary antibody (1:50 dilution) overnight at 4°C. The following day, tissues were washed with DPBS (1x) three times for 5 minutes each wash and then incubated with Alexa Fluor 594 or 647-conjugated secondary antibodies (1:250 dilution) for 30 minutes at room temperature (ThermoFisher Scientific, Waltham, MA). Lastly, tissues were washed with DPBS (1x) before being mounted with ProLong Gold Antifade with 4’6-diamidino-2-phenylindole (DAPI) (Cat#P36931, ThermoFisher Scientific, Waltham, MA). Nail polish was applied around the perimeter of the coverslips to prevent the tissues from drying out. Images were acquired on the EVOS FL microscope using the DAPI, Texas Red, or CY5.5 light cubes at 40x objective (ThermoFisher Scientific, Waltham, MA).

### Mammary gland whole mounts

Mammary glands were fixed in Carnoy’s fixative (six parts 100% ethanol: three parts chloroform: one part glacial acetic acid) for 2 to 4 hours at room temperature. Mammary glands were then washed in 70% ethanol for 20 minutes and changed to decreasing amounts of ethanol and finally to distilled water. The mammary glands were stained overnight in a solution of 0.2% carmine and 0.5% aluminum potassium phosphate (Sigma-Aldrich, St. Louis, MO). Mammary glands were then dehydrated using graded ethanol solutions and left in xylene to clear the fat. Mammary gland whole mounts were stored in methyl-salicylate. Images were captured at 40x using an Olympus DP71 camera.

### Trichrome staining

Masson’s trichrome method for connective tissue (Cat#k037) was adopted from Poly Scientific R&D Corporation (Bayshore, NY). Briefly, tissues were placed in Bouin’s Fixative overnight at room temperature to increase the intensity of the stain. Tissues were washed in running tap water for 10 minutes followed by a distilled water rinse prior to being submerged in Weigert’s Iron Hematoxylin Working Solution for 10 minutes. Tissues were then washed in running tap water for 10 minutes followed by a distilled water rinse prior to being placed in Biebrich Scarlet Acid Fuchsin. Tissues were rinsed twice with distilled water for 15 seconds and subsequently placed in Phosphotungstic Phosphomolybdic Acid for 12 minutes. Tissues were then submerged in Aniline Blue Solution for 20 minutes followed by two 15 second distilled water rinses. Lastly, tissues were placed in 1% Acetic Acid to clear the tissues of any loosely bound dye. Tissues were dehydrated in a series of ethanol solutions, placed in xylene for 5 minutes, and mounted with Permount (ThermoFisher Scientific, Waltham, MA). Images were acquired at 40x using the EVOS XL Core microscope.

### Cell lines

MCF10DCIS.com cells were obtained through a Material Transfer Agreement with the Barbara Ann Karmanos Cancer Institute at Wayne State University. The MCF10DCIS.com cell line was cultured in phenol-free DMEM/F12 medium (Cat#21041025, ThermoFisher Scientific, Waltham, MA) supplemented with 5.26% charcoal-stripped horse serum (Cat#NC9058780, ThermoFisher Scientific, Waltham, MA), 1.05mM calcium chloride (Cat#21115-100ML, Sigma-Aldrich, St. Louis, MO), and 10mM HEPES (Cat#15630080, ThermoFisher Scientific, Waltham, MA). 293T cells (Cat#CRL-3216, ATCC, Manassas, VA) were cultured in DMEM medium (Cat#11965118) supplemented with 10% fetal bovine serum (FBS) (Cat#16140071) and 1% penicillin/streptomycin (Cat#15140163) (ThermoFisher Scientific, Waltham, MA). All cell lines were incubated at 37°C / 5% CO_2_.

### Bacterial transformation and lentiviral transduction

All work involving bacterial transformation and lentiviral transduction was approved by the Institutional BioSafety Committee at University of the Sciences. A STAT5a plasmid (TRCN0000473086, NM_003152.3) and an Empty Vector plasmid (ORFPUR) were purchased from Sigma-Aldrich (St. Louis, MO). Briefly, plasmids were grown up to desired concentrations using GCIL3 ultracompetent bacteria by selecting with ampicillin (50mg/mL). Plasmid DNA was isolated, purified, and precipitated using the PureLink HiPure Plasmid Filter Maxiprep Kit (Cat#K210016, ThermoFisher Scientific, Waltham, MA). Next, 293T packaging cells were co-transfected with the plasmids and the Lenti-Pac HIV Expression Packaging Kit (Cat#LT001, GeneCopoeia, Rockville, MD) to produce lentiviral particles. Lastly, MCF10DCIS.com cells were infected with 1mL of Empty Vector or STAT5a Overexpressor lentiviral particles in complete medium supplemented with 5μg/mL Polybrene for 24 hours (Cat#sc-134220, Santa Cruz Biotechnology, Santa Cruz, CA). Stable cell lines were generated upon selection with 10μg/mL puromycin dihydrochloride for two weeks (Cat#108071, Santa Cruz Biotechnology, Santa Cruz, CA).

### Immunofluorescence on cells

Briefly, 2.0 x 10^5^ cells were seeded on poly-l-lysine coated coverslips (Cat#P4707, Sigma-Aldrich, St. Louis, MO) in 6 well plates (Cat#3516, Corning, Corning, NY). The following day, cells were fixed in 4% paraformaldehyde (Cat#159-SP, Electron Microscopy Sciences, Hatfield, PA) for 15 minutes at room temperature. Fixed cells were washed three times with DPBS (1x) and then permeabilized in 100% methanol for 10 minutes at -20°C. Following another DPBS (1x) wash, cells were blocked in blocking buffer (DPBS 1x, 5% normal serum, 0.3% Triton X-100) for 60 minutes at room temperature. Cells were then incubated with a given primary antibody (1:50 dilution in DPBS 1x, 1% BSA, 0.3% Triton X-100) for 1 hour at 37°C. After three DPBS (1x) washes, cells were incubated with an Alexa Fluor 594-conjugated goat anti-rabbit secondary antibody (1:500 dilution in DPBS 1x, 1% BSA, 0.3% Triton X-100) for 1 hour at 37°C (Cat#A-11012, ThermoFisher Scientific, Waltham, MA). Cells were washed three times with DPBS (1x) before being mounted with ProLong Gold Antifade with DAPI (Cat#P36931, ThermoFisher Scientific, Waltham, MA). Using the EVOS FL microscope, images were acquired at 40x objective using the DAPI and Texas Red light cubes.

### Transwell invasion assay

Briefly, 1.5 x 10^5^ cells were resuspended in 500uL of phenol-free growth medium containing a reduced serum concentration of 0.5% charcoal-stripped horse serum. Culture medium was supplemented with vehicle (DMSO) or 1nM estradiol (Cat#E1024-1G, Sigma-Aldrich, St. Louis, MO). Cells were then seeded in invasion chambers with membranes (8μm pore size) pre-coated with growth factor reduced matrigel (Cat#354483, Corning, Corning, NY). Invasion chambers were placed in 24 well plates (Cat#353047, Corning, Corning, NY), with each well containing 750uL of phenol-free growth medium at a normal serum concentration of 5.26% charcoal stripped horse serum. Chambers were incubated at 37°C/5% CO_2_ for 18 hours to allow cells to invade. The following day, the inner membranes of the chambers were washed with PBS (1x) and gently scraped with cotton swabs to remove any non-invaded cells and the matrigel layer. Invaded cells located on the outer membrane were fixed with 100% methanol at room temperature for 10 minutes, rinsed with distilled water, and left to dry before being mounted with Vectashield mounting medium with DAPI (Cat#H-1200, Vector Laboratories, Burlingame, CA). Using the EVOS FL microscope, five representative fields of view of the DAPI-stained invaded cells on each membrane at captured at 10x objective (ThermoFisher Scientific, Waltham, MA).

### Western blot

Samples were homogenized in RIPA lysis buffer (50mM Tris pH 7.5, 150 mM NaCl, 1% Nonidet P-40, 0.5% deoxycholate, 0.1% SDS) supplemented with complete mini protease inhibitor cocktail (Cat#NC0969110, Roche Diagnostics, Basel, Switzerland) and phosphatase inhibitor cocktail (Cat#78428, ThermoFisher Scientific, Waltham, MA). After homogenization, samples were sonicated and centrifuged at 10,000 x rpm for 10 minutes at 4°C. Supernatant was then collected for measurement of protein concentration using a bicinchoninic acid (BCA) kit (Cat#23225, ThermoFisher Scientific, Waltham, MA). Samples (75μg -100μg) were separated by SDS-PAGE (12% acrylamide) and transferred to a nitrocellulose membrane (Cat#45-004-001, GE Healthcare, Chicage, IL) for probing. Subsequent wash buffers consisted of 10 mM Tris pH 8.0, 150 mM NaCl, 0.05% Tween 20 (TBS-T). Membranes were blocked in TBS-T supplemented with 5% bovine serum albumin (BSA) (Cat#BP1600-100, ThermoFisher Scientific, Waltham, MA) or 5% nonfat dry milk (Cat#50-447-778, Quality Biological Inc., Gaithersburg, MD) for 1 hour at room temperature. The membranes were subsequently incubated with a given primary antibody (1:100 to 1:500 dilution) overnight at 4°C. IRDye 680RD or 800CW secondary antibodies (1:15,000 dilution) were used to visualize bound primary antibodies (LI-COR, Lincoln, NE). The Odyssey CLx Imaging System was utilized for near-infrared (NIR) fluorescent detection of proteins (LI-COR, Lincoln, NE). Image Studio software version 5 on the Odyssey CLx was used to quantify Western bands (LI-COR, Lincoln, NE).

### Statistical analysis

Statistical analyses were performed using Excel and SAS software version 9.4 (SAS Institute Inc., Cary, NC). Differences in ductal branching were evaluated using a one-way ANOVA followed by posthoc Bonferroni adjusted tests for multiple comparison. Differences in ductal foci formation were evaluated using a Kruskal-Wallis test followed by posthoc adjusted tests for multiple comparisons based on the Dwass, Steel, Critchlow-Fligner method. Comparisons involving only two groups were analyzed using a two-tailed t-test. Data were reported as mean +/- standard error of the mean (SEM). Differences in fold change between groups were analyzed using a Wilcoxon two-sample test. Median and interquartile range (IQR) were reported. Statistical significance was reached at p<0.05 (*)(#)(†), p<0.01 (**)(##)(††), and p<0.001 (***)(###)(†††).
